# Comparative Mitogenomic Analysis Reveals Sexual Dimorphism in a Rare Montane Lacewing (Insecta: Neuroptera: Ithonidae)

**DOI:** 10.1371/journal.pone.0083986

**Published:** 2013-12-31

**Authors:** Yuyu Wang, Xingyue Liu, Shaun L. Winterton, Yan Yan, Wencheng Chang, Ding Yang

**Affiliations:** 1 Department of Entomology, China Agricultural University, Beijing, China; 2 California State Arthropod Collection, California Department of Food and Agriculture, Sacramento, California, United States of America; University of California, Berkeley, United States of America

## Abstract

*Rapisma* McLachlan, 1866 (Neuroptera: Ithonidae) is a rarely encountered genus of lacewings found inmontane tropical or subtropical forests in Oriental Asia. In Xizang Autonomous Region (Tibet) of China there are two sympatrically distributed species of *Rapisma*, i.e. *Rapisma xizangense* Yang, 1993 and *Rapisma zayuanum* Yang, 1993, in which *R. xizangense* is only known as male and has dull brownish body and wing coloration, while *R. zayuanum* is only known as female and has bright green body and wing coloration. In order to clarify the relationship between these two species, we determined the complete mitochondrial (mt) genomes of *R. xizangense* and *R. zayuanum* for the first time. The mt genomes are 15,961 and 15,984 bp in size, respectively, and comprised 37 genes (13 protein coding genes, 22 tRNA genes and 2 rRNA genes). A major noncoding (control) region was 1,167 bp in *R. xizangense* and 1,193 bp in *R. zayuanum* with structural organizations simpler than that reported in other Neuropterida species, notably lacking conserved blocks or long tandem repeats. Besides similar mitogenomic structure, the genetic distance between *R. xizangense* and *R. zayuanum* based on two rRNAs and 13 protein coding genes (PCGs) as well as the genetic distance between each of these two Tibetan *Rapisma* species and a Thai *Rapisma* species (*R. cryptunum*) based on partial *rrnL* show that *R. xizangense* and *R. zayuanum* are most likely conspecific. Thus, *R. zayuanum* **syn. nov.** is herein treated as a junior synonym of *R. xizangense*. The present finding represents a rare example of distinct sexual dimorphism in lacewings. This comparative mitogenomic analysis sheds new light on the identification of rare species with sexual dimorphism and the biology of Neuroptera.

## Introduction

In many groups of animals sexual dimorphism results in distinct variation in body size, coloration, and certain non-genitalic structures between males and females in the same species, beyond the intersexual differences of the genitalic structures [1], [2]. Sexual dimorphism is considered to be the result of the sexual selection in the long evolutionary progress [2]. Sexual dimorphism are found in many animals such as the macaques [3], the Chinese mitten crabs [4], and the poison frogs [5]. Sexual dimorphism has also been observed in at least 23 orders of Insecta and it is frequently reported in Phasmida, Orthoptera, Hemiptera, Coleoptera, Diptera, and Lepidoptera [6]. For example, stick insects of *Timema cristinae* have rather different body size and coloration between males and females than between populations [7]. The male stag beetles (Coleoptera: Lucanidae) often have enlarged mandibles, which present as a weapon for male-to-male combat [8]. Sexual dimorphism can sometimes result in difficulties in identifying the different sexes of a particular species, with many examples of males and females of a species being described in different species or even genera, which is usually present, for example, in the taxonomy of stag beetles [9].

Neuroptera (lacewings) are an order of Neuropterida with approximately 6000 extant species described in 17 families (Nevrorthidae, Sisyridae, Osmylidae, Hemerobiidae, Chrysopidae, Ithonidae, Polystoechotidae, Coniopterygidae, Dilaridae, Berothidae, Rhachiberothidae, Mantispidae, Psychopsidae, Nemopteridae, Nymphidae, Myrmeleontidae, and Ascalaphidae) world-wide [10]. Sexual dimorphism is rare in Neuroptera, but is recorded in e.g. Dilaridae, in which the male has pectinate antennae while the female has filiform or moniliform antennae, and in Coniopterygidae, in which there are brachypterous and apterous females in certain species, and there are different arrangements of scale-like bristles on wings and thorax in males and females of many Berothidae. Another sexual dimorphism is to be found in the Crocinae (Nemopteridae) with respect to the bulla in the wings of males. One of the most spectacular dimorphisms in Neuroptera is the marked difference of the coloration of the male and female wings in *Dimares elegans* (Perty, 1833) (Myrmeleontidae). Furthermore, the male adults are relatively smaller than females in most species of Neuroptera, but they have similar body and wing coloration, therefore making some difficulties in distinguishing males and females by external appearance.

Rapismatidae (montane lacewings) contains ca. 20 species endemic to the Oriental realm, all placed in the genus *Rapisma* McLachlan. The group was elevated to family level status by Navás [11], despite remarkable similarity to members of the family Ithonidae (moth lacewings), and a lack of well defined synapomorphies unique to the family. The family level status of Rapismatidae has been supported subsequently by various authors [12]–[15] but questioned by other authors [16]–[22], most including *Rapisma* in Ithonidae. Barnard [12] identified a series of synapormophies unique to Rapismatidae, but were subsequently shown by Penny [17] to be either plesiomorphic or too variable to support continued use of Rapismatidae. In quantitative phylogenetic analyses of relationships using DNA sequences and morphology, Haring & Aspöck [23], Winterton *et al.* [24] and Winterton & Makarkin [19] identified the clear paraphyly of Ithonidae relative to Polystoechotidae (giant lacewings) and in the latter provided clear evidence for the synonymy of both Polystoechotidae and Rapismatidae with Ithonidae. Adult Ithonidae are characterized by the medium to large sized robust body with small head retracted under the pronotum, short antennae, and broad wings with recurrent humeral veinlets, which are pectinate in both fore- and hindwings. The larvae of Ithonidae are known for a few genera in Australia and North America but are unknown for *Rapisma*. Ithonidae larvae are typically robust, scarabaeiform and fossorial, presumably feeding on root exudates [16], [25], [26]. In southwestern China there are four described montane lacewing species, namely *Rapisma daianum* Yang, 1993 from Yunnan, *Rapisma xizangense* Yang, 1993 from Xizang (Tibet), *Rapisma yanhuangi* Yang, 1993 from Sichuan, and *Rapisma zayuanum* Yang, 1993 from Xizang (Tibet) [13]. Among the four Chinese species, *R. xizangense* and *R. zayuanum* are sympatrically distributed in southeastern Xizang, with both holotypes collected from same locality (i.e. Xizang, Zayu County, Gyiang, alt. 2300–2400 m). Interestingly, *R. xizangense* is only known as male, which is generally dull brownish, while *R. zayuanum* is only known as female, which is generally bright green and much larger than the male of *R. xizangense*.

Sexual dimorphism in *Rapisma* was discussed by Barnard & New [27], [28] based on the intersexual comparison for two species, i.e. *Rapisma burmanum* Navás, 1929 and *Rapisma berhalense* Barnard & New, 1985 (all other species of *Rapisma* are known as only male or female). Differentiating traits between males and females of *Rapisma* are considered to be the relative size of compound eyes (i.e. EI ratio: ratio between maximum eye diameter and minimum inter-ocular distance) and the shape of forewings. In observed males of *Rapisma*, the EI ratio is higher than that in females, and the forewing has a relatively narrow costal region [27], [28]. However, the coloration between males and females of *R. burmanum* and *R. berhalense* are quite similar, generally yellow to yellowish brown.

Recently, we obtained one male and one female specimen of *Rapisma* collected from same locality in Zayu, southeastern Xizang. Based on the morphological identification and quite close geographical distribution compared with *R. xizangense* and *R. zayuanum*, the male specimen was identified to be *R. xizangense* and female specimen was identified to be *R. zayuanum*. Except for these two sympatrically distributed species from Xizang, all other *Rapisma* species are distributed allopatrically [12], [27], which raises a question that whether *R. xizangense* and *R. zayuanum* are conspecific. Due to the relative rarity of *Rapisma*, we can neither obtain large number of specimens for DNA barcoding test nor directly observe the copulation.

In this paper, we determine the complete mitochondrial genomes of *R. xizangense* and *R. zayuanum*. We calculate the genetic distance of 15 genes between the two species as well as an additional *Rapisma* species from Thailand. We also compared the genomic structure and composition, such as gene content, RNA secondary structure, and gene order, with other Neuropterida species (four species of Megaloptera, seven species of Neuroptera and one species of Raphidioptera) with their mt genomes already published [29]–[35]. The mt genomic data confirm that *R. xizangense* and *R. zayuanum* are conspecific but have extraordinarily striking sexual dimorphism in coloration.

## Results

### Genome organization

**Figure 1:**
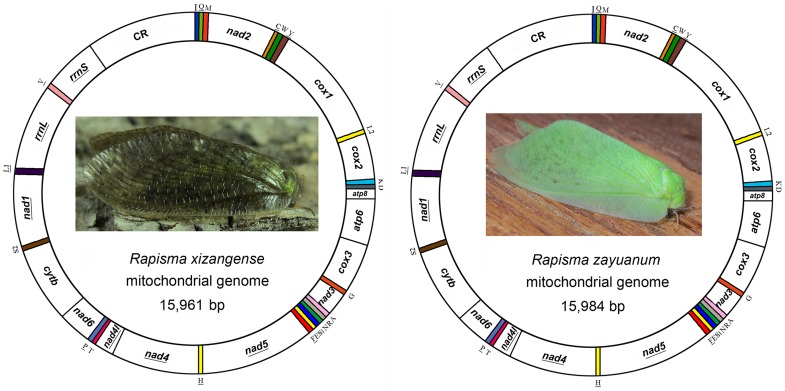
Mitochondrial maps of *Rapisma xizangense* (left) and *Rapisma zayuanum* (right). The tRNAs are denoted by the colour blocks and are labelled according to the IUPACIUB single-letter amino acid codes. Gene name without underline indicates the direction of transcription from left to right, and with underline indicates right to left.

**Figure 2:**
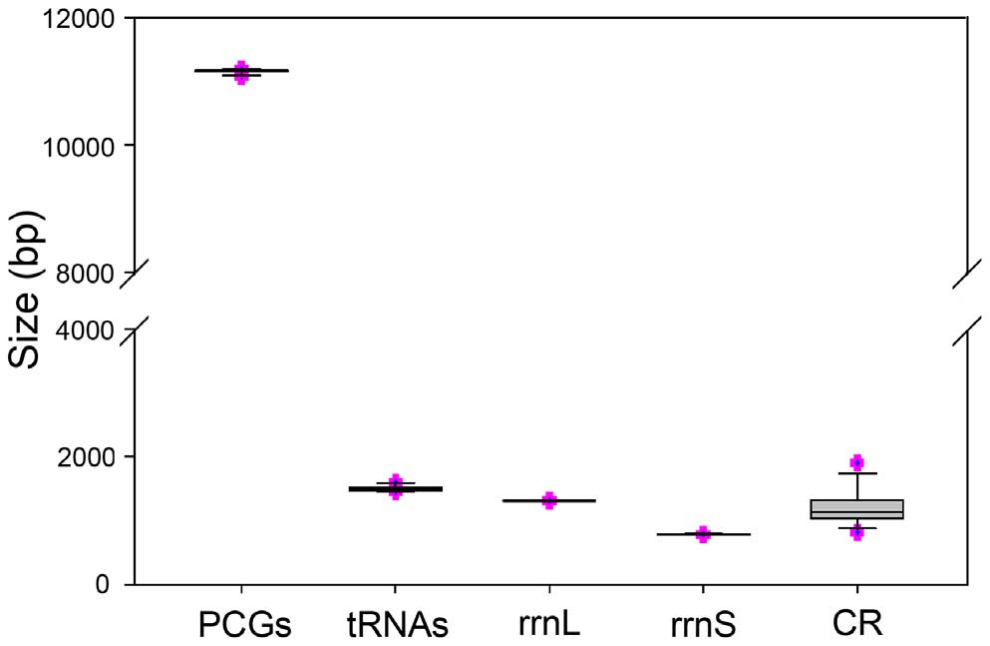
The size of PCGs, *rrnL*, *rrnS*, and CR, respectively, among sequenced Neuropterida mt genomes.

The complete mt genomes of *R. xizangense* (15,961 bp) and *R. zayuanum* (15,984 bp) were determined (GenBank accession number KF626446, KF626447; Figure 1). The composition and structure of these two mt genome are exactly the same. The mt genome of the two insects are medium-sized when compared with those of other Neuroptera species, whose mt genomes typically range from 15,791 bp to 16,723 bp and are relatively larger than those in Megaloptera and Raphidioptera. Among Neuropterida mt genomes, the length variation is minimal in PCGs, tRNAs, *rrnL* and *rrnS*, but very different in the putative control region (Figure 2; Table S2). The mt genomes of *R. xizangense* and *R. zayuanum* contain all 37 genes (13 PCGs, 22 tRNA genes, and 2 rRNA genes) that are typically present in metazoan mt genomes [36]. The gene order of the two mt genomes slightly differ from the putative insect ancestral gene order of *Drosophila yakuba* (Burla) [37] but conforms to the presumed synapomorphy of Neuroptera, in that the *tRNA^Cys^* is located upstream rather than its traditional downstream location relative to *tRNA^Trp^* [29], [32]–[35], [38]. The mt genomes of *R. xizangense* and *R. zayuanum* both contain 10 small non-coding regions, ranging in size from 1 to 29 bp. These were distributed among PCGs, tRNAs, *rrnL* and *rrnS* (Table S3). The control region of *R. xizangense*, between the *rrnS* and *tRNA^Ile^*, occupies 1167 bp, while the control region of *R. zayuanum* occupies 1193 bp. The control regions of the two insects both have simple structure compared with other Neuroptera species without conserved blocks and long tandem repeats. The A+T composition in this region of *R. xizangense* and *R. zayuanum* respectively is 94.94% and 94.89%, much higher than that of the coding region. Twenty-three genes were transcribed on the majority strand (J-strand), whereas fourteen genes were oriented on the minority strand (N-strand). Gene overlaps were found at 11 gene junctions and involved a total of 35 bp; the longest overlap (8 bp) existed between *nad6* and *cytb*.

### Base composition and codon usage

**Figure 3:**
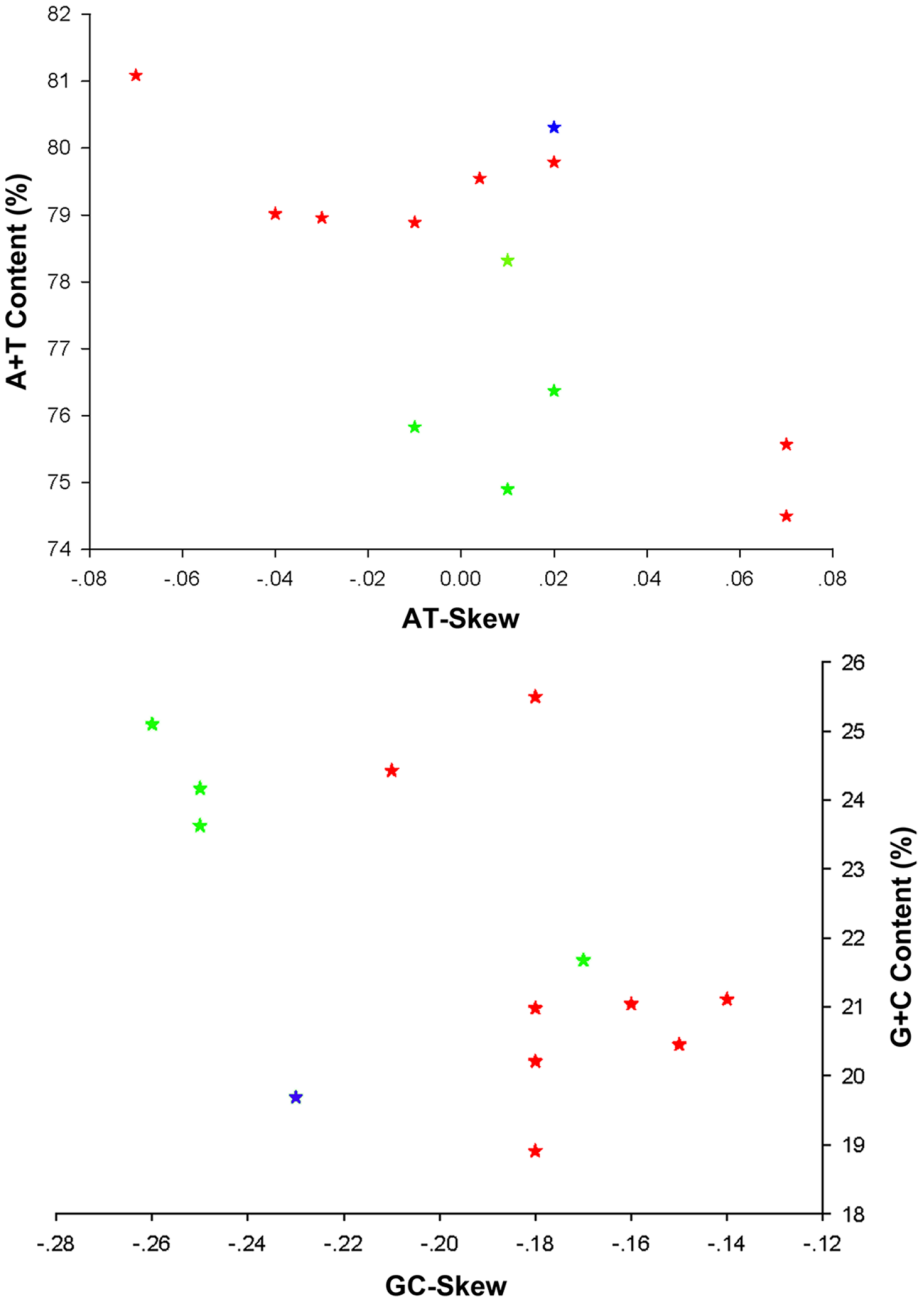
AT% vs AT-Skew and GC% vs GC-Skew in Neuropterida mt genomes. Measured in bp percentage (Y-axis) and level of nucleotide skew (X-axis). Values are calculated on full length mt genomes. Blue pentacle, Raphidioptera; red pentacle, Neuroptera; green pentacle, Megaloptera.

Similar to mt genome sequences of other Neuropterida species, the nucleotide composition of the *R. zayuanum* mt genome is also biased toward A and T (A = 37.81%, T = 43.28%, G = 7.71%, C = 11.20%; Table S4). The overall A+T content (81.09%) of *R. zayuanum* is the highest among the Neuropterida mt genomes (Table S4). The metazoan mt genomes usually present a clear strand bias in nucleotide composition [39], [40], and the strand bias can be measured as AT- and GC-skews [41]. A comparative analysis of A+T% vs AT-skew and G+C% vs GC-skew across all available mt genomes of Neuropterida is shown in Figure 3. The average AT-skew of the Neuropterida mt genomes is 0.005, ranging from −0.070 in *R. zayuanum* to 0.070 in two owlfly species (*Libelloides macaronius* (Scopoli, 1763) and *Ascaloptynx appendiculatus* Fabricius, 1793), indicating that the mt genome of *R. zayuanum* exhibits a strong AT-skew (Table S4). Whereas, the average GC-skew of Neuropterida mt genomes was −0.195, ranging from −0.260 in a dobsonfly *Corydalus cornutus* (Linnaeus, 1758) to −0.140 in a green lacewing *Chrysoperla nipponensis* Okamoto, 1914 and the mt genome of *R. zayuanum* exhibits a weak GC-skew (−0.180) (Table S4).

**Figure 4:**
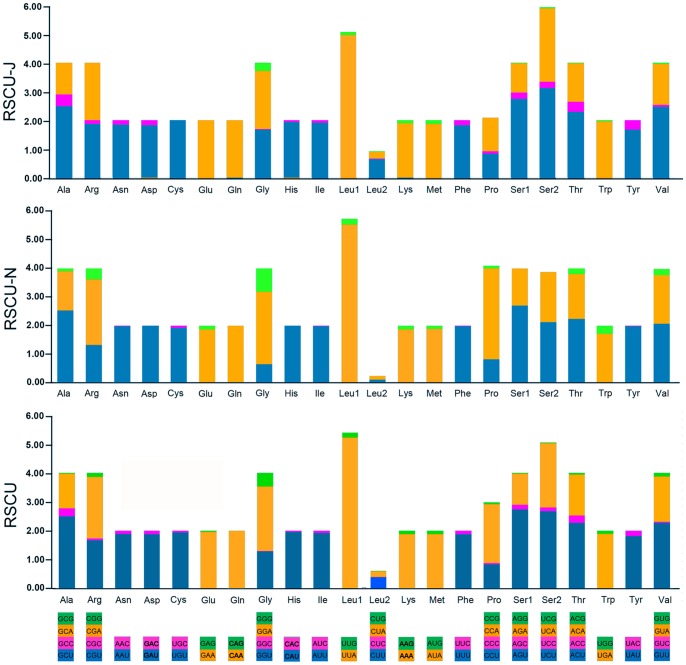
Relative synonymous codon usage (RSCU) in the *Rapisma zayuanum* mt genome. Codon families are provided on the x-axis.

The 13 PCGs exhibit the canonical mitochondrial start codons for invertebrate mtDNAs [36], TTG for the *nad1* and ATN for the remaining 12 PCGs. Stop codons for the PCGs were almost invariably complete TAA or incomplete T except TAG for nad1. The genome-wide bias toward AT was well documented in the codon usage (Table S5). At the third codon position, A or T were overwhelmingly represented compared to G or C. The overall pattern is very similar among the mt genomes of the Neuropterida species, with similar frequency of occurrence of various codons within a single codon family. There is a strong bias toward AT-rich codons with the six most prevalent codons in *R. zayuanum*, as in order, TTA-Leu (14.25%), ATT-Ile (11.78%), TTT-Phe (9.19%), ATA-Met (5.87%), AAT-Asn (5.82%), and TAT-Tyr (4.39%), (Table S5). Six most frequently used codons above were all composed wholly of A and/or T (Figure 4). The base compositions and codon usage of *R. xizangense* is almost the same with *R. zayuanum*.

### Protein-coding genes

The total length of all 13 PCGs was 11,164 bp, accounting for 69.84% of the entire length of *R. zayuanum* mt genome. The overall AT content of PCGs was 79.30%, ranging from 73.06% (*cox1*) to 88.68% (*atp8*) (Table S6). Start and stop codons were determined based on alignments with the corresponding genes of other Neuroptera species. Five genes (*atp6, cox2*, *cox3, cytb, nad4*) use the standard ATG start codon, four genes (*atp8*, *cox1, nad2*, *nad3*) initiate with ATT, three genes (*nad4l, nad5, nad6*) start with ATA, and *nad1* initiates with TTG. Nine genes employ a complete translation termination codon, either TAG (*nad1*) or TAA (*atp6, atp8*, *cox3, cytb, nad2, nad3, nad4l, nad6*,), whereas the remaining four have incomplete stop codons T (*cox1, cox2*, *nad4, nad5*). The absence of some G+C-rich codons was found in *R. zayuanum* and the codon CAG was not used.

Sequence overlaps were found between 11 neighbour genes (Table S3). In the *R. zayuanum* mt genome, the overlap nucleotides were conserved (ATGATAA for *atp8/atp6* and ATGTTAA for *nad4l/nad4*). These overlapped sequences were also observed in other four species of Megaloptera (*C. cornutus*, *Protohermes concolorus* Yang & Yang, 1988 *Sialis hamata* Ross, 1937 and *Neochauliodes punctatolosu*s Liu & Yang, 2006) as well as three species of Neuroptera (*Polystoechotes punctatus* (Fabricius, 1793), *Chrysopa pallens* (Rambur, 1838) and *Ditaxis biseriata* (Westwood, 1852)). The protein-coding genes of *R. xizangense* are the same with *R. zayuanum* in length, start codons, stop codons as well as sequence overlaps.

**Figure 5:**
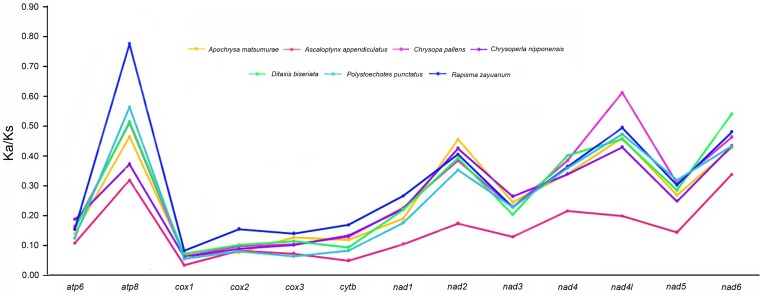
Evolution rates of the 13 protein-coding genes of Neuroptera.

The rate of non-synonymous substitutions (Ka), the rate of synonymous substitutions (Ks), and the ratio of Ka/Ks were calculated for each PCG of seven species of Neuroptera (Table S7). The reference sequence is the *L. macaronius*. In this respect, *atp8* shows the highest evolutionary rates 0.77 in *R. zayuanum*, followed by *nad4l* 0.61 in *C. pallens*. The ratio of Ka/Ks for each species and every PCG is below 1, indicating that these genes are evolving under the purifying selection [42], [43] (Figure 5).

### Transfer RNAs

**Figure 6:**
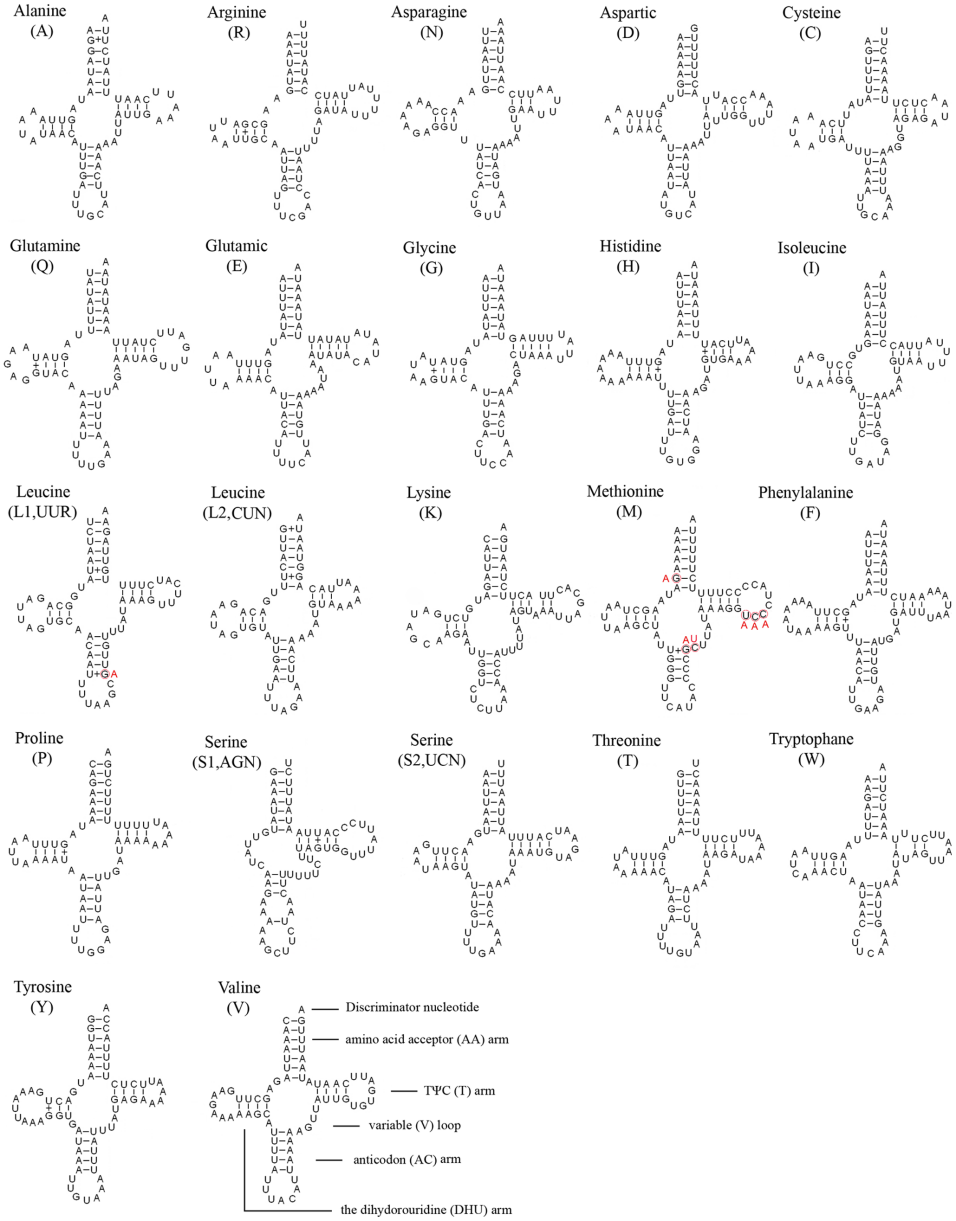
Inferred secondary structure of 22 tRNAs of *Rapisma xizangense* and *Rapisma zayuanum*. The tRNAs are labelled with the abbreviations of their corresponding amino acids. Inferred Watson-Crick bonds are illustrated by lines, whereas GU bonds are illustrated by dots. The red letters indicate the mutations in *R. xizangense*.

The entire complement of 22 typical tRNAs in the arthropod mt genomes was found in *R. zayuanum* and schematic drawings of their respective secondary structures are shown in Figure 6. Most of the tRNAs could be folded as classic clover-leaf structures, with the exception of *tRNA^Ser(AGN)^*, in which its DHU arm simply forms a loop, which is a common phenomenon in sequenced Neuropterida mt genomes. Within the 22 tRNA genes, 14 genes were encoded by the J-strand, while the remains were coded by the N-strand.

The length of tRNAs ranged from 63 to 71 bp. The aminoacyl (AA) stem (7 bp) and the AC loop (7 nucleotides) are invariable, with the exception of *tRNA^Thr^* AC loop (9 nucleotides). The DHU and TΨC (T) stems are variable while the loop size (4–9 nucleotides) was more variable than the stem size (0–5 bp). The size of the anticodon (AC) stems was constantly 5 bp, except the *tRNA^Lys^, tRNA^Met^, tRNA^Ser(AGN)^* and *tRNA^Thr^* whose AC stem size was 4 bp. Based on the secondary structure, 15 mismatched base pairs were found in *R. zayuanum* tRNAs. All of them were G-U pairs located in the AA stem (4 bp), the DHU stem (7 bp), the AC stem (2 bp), the T stem (2 bp).

The tRNAs of the *R. xizangense* is exactly the same with those of *R. zayuanum* except *tRNA^Leu(UUR)^* and *tRNA^Met^*. As to *tRNA^Leu(UUR)^*, there is a U+G base pair in the AC arm of *R. zayuanum*, while it is U-A base pair of *R. xizangense*. The *tRNA^Met^*, in the same way, there is a G-C base pair in the AA arm, a U+G base pair in the AC arm, a C in the V loop, and a UCC in the T arm of *R. zayuanum*; while it is a AC mismatch, a U-A base pair, a U and a AAA of *R. xizangense* respectively (Figure 6).

### Ribosomal RNAs

**Figure 7:**
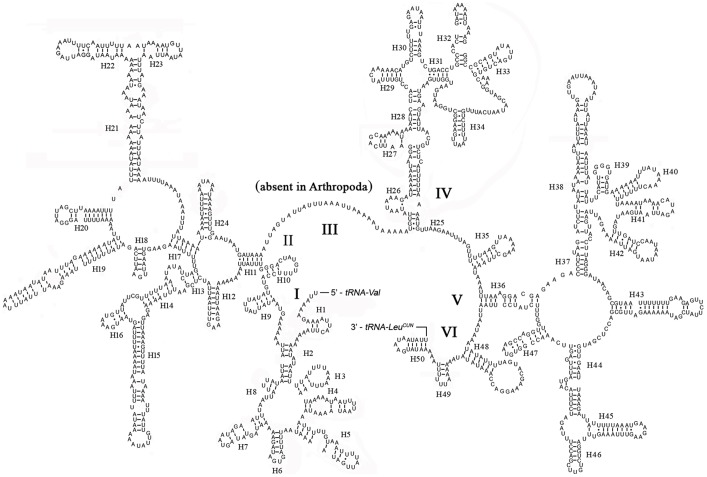
Predicted secondary structure of the *rrnL* gene in *Rapisma zayuanum*. Inferred Watson-Crick bonds are illustrated by lines, GU bonds by dots.

**Figure 8:**
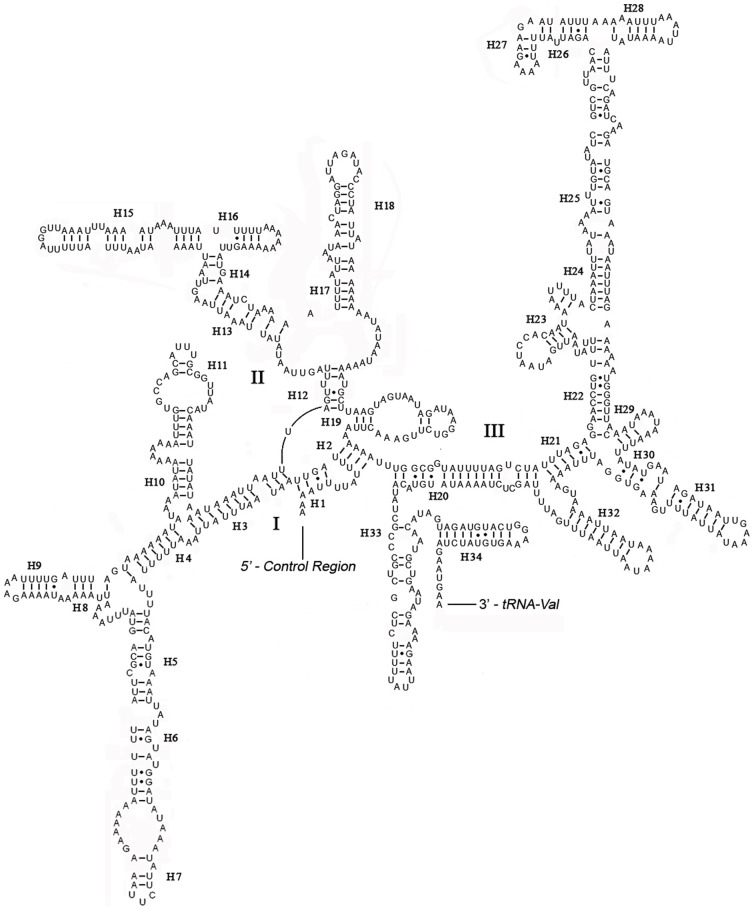
Predicted secondary structure of the *rrnS* gene in *Rapisma zayuanum*. Roman numerals denote the conserved domain structure. Inferred Watson-Crick bonds are illustrated by lines, GU bonds by dots.

The boundaries of rRNA genes were determined by sequence alignment with other Neuroptera species. The *rrnL* was assumed to fill up the blanks between *tRNA^Leu(CUN)^* and *tRNA^Val^* and the *rrnS* to fill up the blanks between *tRNA^Val^* and the non-coding putative control region [29], [30], [32]. The length of *rrnL* and *rrnS* of *R. zayuanum* was determined to be 1,316 bp and 777 bp, respectively. Both *rrnL* and *rrnS* are generally congruent with the secondary structure models proposed for other insects [34], [44]–[47]. The structure of *rrnL* of *R. zayuanum* largely resembles previously published structures for *L. macaronius* [34], and the inferred secondary structure presents five canonical domains (I–II, IV–VI) with domain III absent, which is a typical trait in arthropods [44] (Figure 7), and includes 50 helices. The highest level of invariable positions was located on domain IV, while lowest level was on domains I–II. The *rrnS* of *R. zayuanum* is largely in agreement with those proposed for other holometabolous orders, including three domains and 34 helices (Figure 8). The *rrnL* and *rrnS* of *R. xizangense* are almost the same in both the length and the proposed secondary structures.

### Phylogenetic analysis

**Figure 9:**
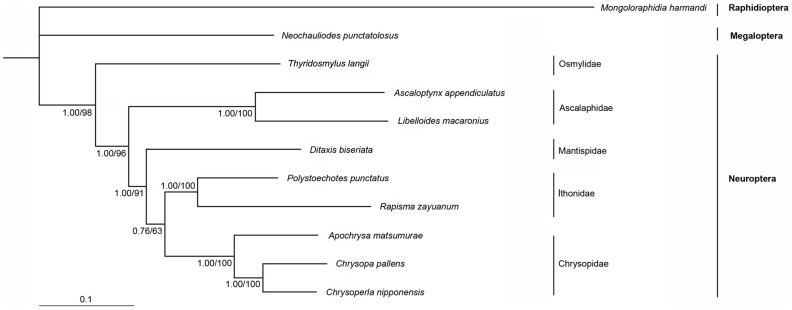
Phylogenetic relationships among the Neuroptera insects based on mt genomes. Numbers at the nodes are Bayesian posterior probabilities (left) and ML bootstrap values (right).

In the phylogenomic analysis on the higher relationships within Neuroptera, the phylogenetic trees generated from Bayesian and ML inferences have the same topologies based on the PCG123R matrix (totally 12729 sites) (Figure 9). The interfamilial relationships are consistent with the previous result from Wang *et al.* [31]. In addition, Osmylidae is assigned to be the sister to all other neuropteran taxa. *Rapisma* is the sister of *Polystoechotes*, supporting the monophyly of Ithonidae.

### Genetic distance among *Rapisma* spp

**Figure 10:**
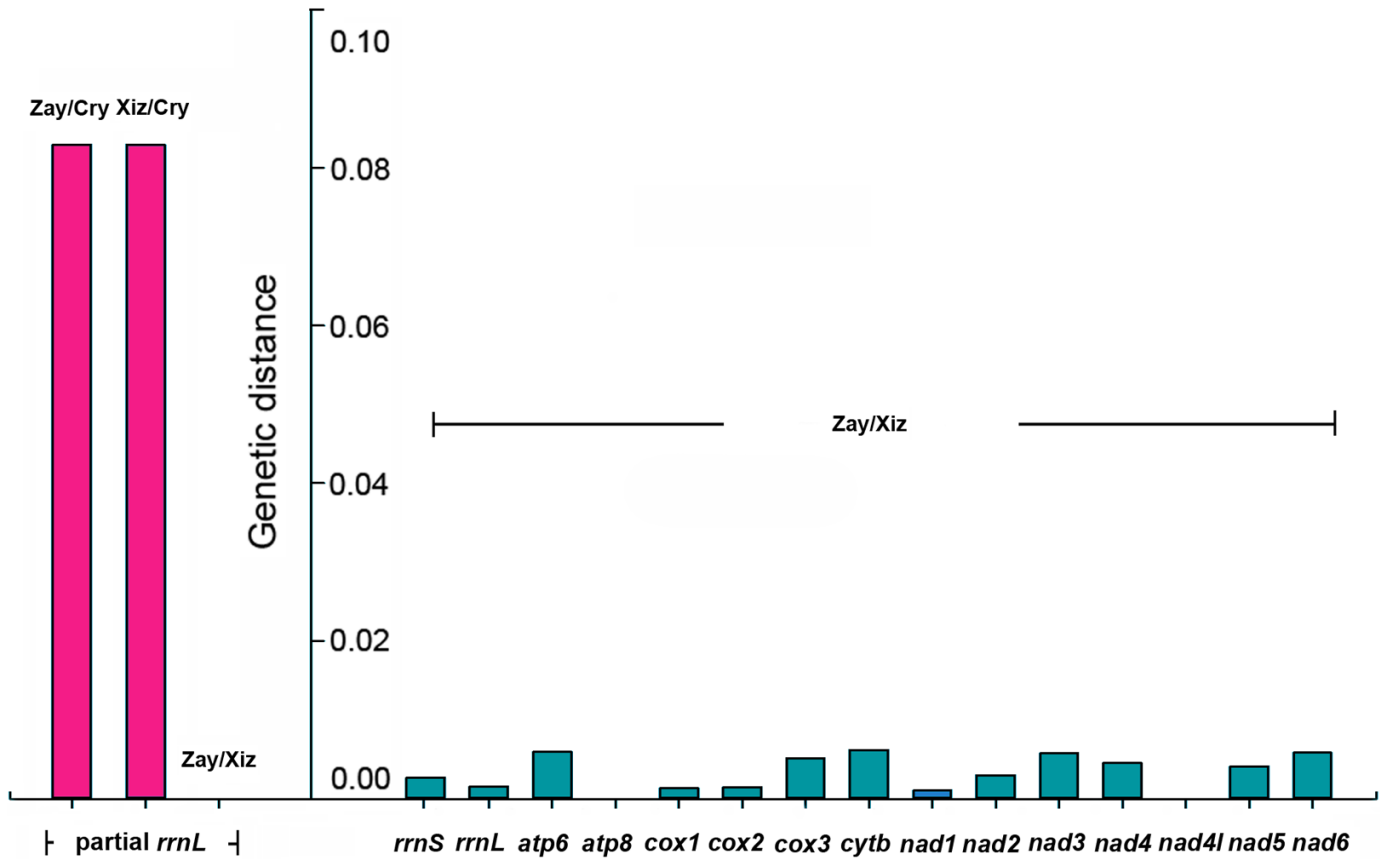
Genetic distance between *Rapisma zayuanum*, *Rapisma xizangense and Rapisma cryptunum* based on partial *rrnL* and genetic distance between *R. zayuanum* and *R. xizangense* based on 13 PCGs, *rrnS*, and *rrnL*.

The genetic distance between *R. zayuanum* and *R. xizangense* is 0.0000 based on partial *rrnL* sequence, while the genetic distance between *R. zayuanum* and *R. cryptunum* or *R. xizangense* and *R. cryptunum* is 0.0829 (Table S8). Moreover, the genetic distance between *R. zayuanum* and *R. xizangense* based on two rRNAs and 13 PCGs (Table S9) ranged from 0.0000 (in *atp8* and *nad4l*) to 0.0062 (in *cytb*). The average genetic distance of the two rRNAs and 13 PCGs is 0.0032, while the average genetic distance of the 13 PCGs is 0.0034 and the average genetic distance of the two rRNAs is 0.0021. Consequently, the genetic distance between *R. zayuanum* and *R. xizangense* is much smaller than that between *R. zayuanum* and *R. cryptunum* or *R. xizangense* and *R. cryptunum* (Figure 10).

## Discussion

### Synonymy of R. xizangense and R. zayuanum

Genetic distance refers to the genetic divergence between species or between populations within a species, and can be used to compare the genetic similarity between different species as well as to measure the divergence between different subspecies. Generally, smaller genetic distances indicate a closer genetic relationship, whereas large genetic distances indicate a more distant genetic relationship. However, the criteria for distinguishing interspecific divergence by using genetic distance are not constant among different molecular markers and taxa. Considering the frequently used molecular marker for DNA barcoding of insects is COI (3′ end), for example, Park *et al.* [48] sequenced the barcodes of COI for 344 true bug species in 178 genera and revealed less than 0.02 intraspecific divergence in 90% of the taxa examined but minimum interspecific distances exceeding 0.03 in 77% of congeneric species pairs. Whereas, Huemer & Hebert [49] presented the barcodes for 766 specimens of 597 lepidopteran species and found that the medium distance to the nearest neighbour is 0.0806 in the fully barcoded species while intraspecific divergence was relatively low with a mean distance of 0.0026. Lourenco *et al.* [50] computed the *p*-distance of *Chrysoperla carnea* group in Europe based on the partial sequences of the mt genes *cytb*, *cox1*, *cox2* and *rrnL*, in which the intraspecific distance are small, ranging from 0.000 to 0.011 in *rrnL*, 0.014 in *cox1* and *cox2*, 0.026 in *cytb*. Herein, the genetic distance of two rRNAs and 13 PCGs between *R. xizangense* and *R. zayuanum* is apparently smaller than 0.01, which indicates likely intraspecific divergence in Neuroptera.

Considering the structure characters of mt genome, the tRNAs of the *R. xizangense* is exactly the same with those of *R. zayuanum* except *tRNA^Leu(UUR)^* and *tRNA^Met^*. As to *tRNA^Leu(UUR)^*, there is only one nucleotide difference in the AC arm of *R. xizangense* and *R. zayuanum*. In *tRNA^Met^*, there are two base pairs and four nucleotides differences in the AA arm, AC arm, V loop and T arm of *R. xizangense* and *R. zayuanum* respectively (Figure 6).

**Figure 11:**
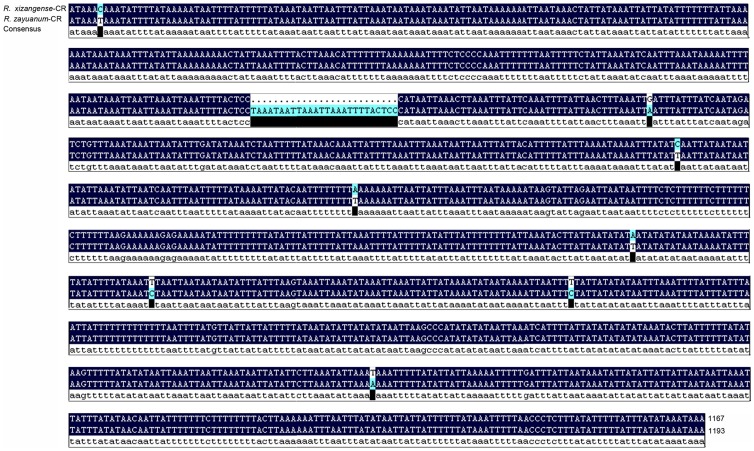
Control region alignment of *Rapisma xizangense* and *Rapisma zayuanum*.

The control regions of the *R. xizangense* and *R. zayuanum* are also almost the same with only few mutations, however, there are 26 nucleotides absent in the control region of *R. xizangense* (Figure 11).

Based on considerably small genetic distance and similar mt genomic structure between *R. xizangense* and *R. zayuanum*, we consider the two species to be conspecific. As the *R. xizangense* was originally described based on a male specimen while in the same paper *R. zayuanum* was described based on only one female specimen [13], *R. zayuanum* is treated as a junior synonym of *R. xizangense* which has better specific definition by having male as holotype.

### Sexual dimorphism of *R. xizangense*

Sexual dimorphism of body and wing coloration is rarely found in Neuroptera and here is reported in Ithonidae for the first time. *Rapisma* adults are nocturnal, while during the daytime adults quietly stay on trees [12], [13]. The *Rapisma* adults are poor fliers [12], [13] and easily found by predators if they lose the protection from their inhabiting environments. Considering *R. xizangense*, males might benefit from their dull brownish coloration when they rest on bark of trees, similarly, females might hide themselves well on foliage. It is difficult to explain why females of *R. xizangense* have bright green coloration but the males are dull brownish, and any biological or behavioural significance for different colour forms. A female of a Thai species, *Rapisma intanonum* Barnard, 1981, with bright green coloration, was reported to rest on a leaf, indicating that the preference of leaf may be common phenomenon for females of *Rapisma* [12].

## Materials and Methods

### Ethics Statement

No specific permits were required for the insects collected for this study in Xizang Autonomous Region. The specimens were collected by using light trap. The field studies did not involve endangered or protected species. The species in the genus *Rapisma* are not included in the “List of Protected Animals in China”.

Samples and DNA extraction

The *R. xizangense* and *R. zayuanum* specimens used to determine the mt DNA were collected from Shaqiong Village, Xiazayu of Zayu County, Xizang Autonomous Region, China, in July 2011. After collection, they were initially preserved in 95% ethanol in the field, and transferred to −20°C for the long-term storage upon the arrival at the China Agricultural University (CAU). Total DNA was purified from muscle tissues of the thorax using TIANamp Genomic DNA Kit (TIANGEN). The quality of DNA was assessed through electrophoresis in a 1% agarose gel and staining with Gold View (nucleic acid stain replacing EB).

### PCR amplification and sequencing

The mt genomes of *R. xizangense* and *R. zayuanum* were generated by amplification of overlapping PCR fragments (Figure 1 and Table S10). Firstly, fourteen fragments were amplified using the universal primers [51]. Then, five specifically designed primers (Table S10) based on the known sequences were used for the secondary PCRs.

All PCRs used NEB Long Taq DNA polymerase (New England BioLabs, Ipswich, MA) under the following amplification conditions: 30 s at 95°C, 40 cycles of 10 s at 95°C, 50 s at 48–55°C, 1 kb/min at 65°C depending on the size of amplicons, and the final elongation step at 65°C for 10 min. The quality of PCR products were evaluated by agarose gel electrophoresis.

All fragments were sequenced in both directions using the BigDye Terminator Sequencing Kit (Applied Bio Systems) and the ABI 3730XL Genetic Analyzer (PE Applied Biosystems, San Francisco, CA, USA) with two vector-specific primers and internal primers for primer walking.

### Bioinformatic analysis

The complete mt genomes of *R. xizangense* and *R. zayuanum* have been deposited in GenBank under accession numbers KF626446 and KF626447. Mt DNA sequences were proof-read and aligned into contigs in BioEdit version 7.0.5.3 [52]. Sequence analysis was performed as follows. Firstly, the tRNA genes were identified by tRNAscan-SE Search Server v.1.21 [53] using invertebrate mitochondrial predictors with a COVE cutoff score of 1, or by sequence similarity to tRNAs of other Neuropterida species. PCGs were identified as open reading frames corresponding to the 13 PCGs in metazoan mt genomes. The rRNA gene boundaries were interpreted as the end of a bounding tRNA gene and by alignment with other Neuropterida gene sequences. The base composition, codon usage, genetic distance and nucleotide substitution were analyzed with MEGA 4.0 [54]. The GC and AT asymmetry was measured in terms of GC and AT skews using the following formulae: AT-skew = (A−T)/(A+T) and GC-skew = (G−C)/(G+C) [41]. Secondary structures of the small and large subunits of rRNAs were inferred using models predicted for *Drosophila yakuba* [37], *Apis mellifera* [45], and *Libelloides macaronius* [34]. Stem-loops were named with Roman numbers. The software packages DnaSP 5.0 [55] was used to calculate the number of synonymous substitutions per synonymous site (Ks) and the number of nonsynonymous substitutions per nonsynonymous site (Ka) for each PCG.

### Phylogenetic analysis

In the phylogenomic analysis on the higher relationships within Neuroptera, the ingroup taxa include nine species of Neuroptera representing five families (Table S1). *Neochauliodes punctatolosus* (Megaloptera) and *Mongoloraphidia harmandi* (Raphidioptera) were selected as outgroups.

DNA alignment was inferred from the amino acid alignment of PCGs using Clustal X [56]. rRNAs alignment was conducted by G-blocks Server (http://molevol.cmima.csic.es/castresana/Gblocks_server.html) by more stringent selection. MrBayes Version 3.1.2 [57] and a PHYML online web server [58], [59] were employed to reconstruct the phylogenetic trees. Model selection was based on Modeltest 3.7 [60] for nucleotide sequences. According to the Akaike information criterion, the GTR+I+G model was optimal for analysis with nucleotide alignments. In Bayesian inference, two simultaneous runs of 2,000,000 generations were conducted. Each set was sampled every 200 generations with a burnin of 25%. Trees inferred prior to stationarity were discarded as burnin, and the remaining trees were used to construct a 50% majority-rule consensus tree. In the ML analysis, the parameters were estimated during analysis and the nodal support values were assessed by bootstrap re-sampling (BP) [61] calculated using 100 replicates. The PCG123R matrix (containing all three codon positions of PCGs, plus the two rRNA genes) was used to reconstruct the phylogeny trees.

## Supporting Information

Table S1 **Summary of taxonomic groups used in this study.**
(DOC)

Table S2 **The size of PCGs, tRNAs,** ***rrnL*** **,** ***rrnS*** **, and CR, respectively, among sequenced Neuropterida mt genomes.**
(DOC)

Table S3 **Organization of the** ***Rapisma zayuanum*** **mt genome.**
(DOC)

Table S4 **Base composition and strand bias in the sequenced mt genomes of Neuroptera.**
(DOC)

Table S5 **Codon usage of protein-coding genes in the** ***Rapisma zayuanum*** **mt genome.**
(DOC)

Table S6 **Base composition and strand bias in PCGs of** ***Rapisma zayuanum*** **.**
(DOC)

Table S7 **Evolution rates of the 13 PCGs of eight species of Neuroptera.**
(DOC)

Table S8 **Genetic distance of the three montane lacewing species based on** ***rrnL*** **.**
(DOC)

Table S9 **Genetic distance between** ***Rapisma zayuanum*** **and** ***Rapisma xizangense*** **based on PCGs and rRNAs.**
(DOC)

Table S10 **Primers used in this study.**
(DOC)
