# Supplementary material for: Comparative Mitogenomic Analysis Reveals Sexual Dimorphism in a Rare Montane Lacewing (Insecta: Neuroptera: Ithonidae)
Source: PLoS One. 2013 Dec 31;8(12):e83986. doi: 10.1371/journal.pone.0083986 (PMC3877146; doi:10.1371/journal.pone.0083986)
Supplement: Table S1 — Summary of taxonomic groups used in this study. (DOC) [file pone.0083986.s001.doc]

**Table S1. Summary of taxonomic groups used in this study**

| **Order/suborder** | **Family** | **Species** | **Accession Number** |
| --- | --- | --- | --- |
| Outgroup |  |  |  |
| **Megaloptera** |  |  |  |
|  | Chauliodinae | *Neochauliodes punctatolosus* | NC_018772 |
| **Raphidioptera** |  |  |  |
|  | Raphidiidae | *Mongoloraphidia harmandi* | NC_013251 |
| Ingroup |  |  |  |
| **Neuroptera** |  |  |  |
|  | Ascalaphidae | *Ascaloptynx appendiculatus* | NC_011277 |
|  | Ascalaphidae | *Libelloides macaronius* | NC_015609 |
|  | Chrysopidae | *Apochrysa matsumurae* | NC_015095 |
|  | Chrysopidae | *Chrysoperla nipponensis* | [NC_015093](http://www.ncbi.nlm.nih.gov/nuccore/NC_012838) |
|  | Chrysopidae | *Chrysopa pallens* | [NC_019618](http://www.ncbi.nlm.nih.gov/nuccore/NC_012838) |
|  | Ithonidae | *Polystoechotes punctatus* | NC_011278 |
|  | Ithonidae | *Rapisma Xizangense* | Present study |
|  | Ithonidae | *Rapisma zayuanum* | Present study |
|  | Mantispidae | *Ditaxis biseriata* | NC_013253 |
|  | Osmylidae | *Thyridosmylus langii* | NC_021415 |
