# Supplementary material for: Comparative Mitogenomic Analysis Reveals Sexual Dimorphism in a Rare Montane Lacewing (Insecta: Neuroptera: Ithonidae)
Source: PLoS One. 2013 Dec 31;8(12):e83986. doi: 10.1371/journal.pone.0083986 (PMC3877146; doi:10.1371/journal.pone.0083986)
Supplement: Table S2 — The size of PCGs, tRNAs, rrnL , rrnS , and CR, respectively, among sequenced Neuropterida mt genomes. (DOC) [file pone.0083986.s002.doc]

**Table S2. The Size of PCGs, tRNAs, *rrnL*, *rrnS*, and *CR*, respectively, among sequenced Neuropterida mt genomes**

| **Species** | **PCGs** | **tRNAs** | **rrnL** | **rrnS** | **CR** |
| --- | --- | --- | --- | --- | --- |
| *Polystoechotes punctatus* | 11168.00 | 1505.00 | 1319.00 | 784.00 | 1123.00 |
| *Libelloides macaronius* | 11177.00 | 1470.00 | 1313.00 | 782.00 | 1049.00 |
| *Ascaloptynx appendiculatus* | 11169.00 | 1520.00 | 1299.00 | 779.00 | 1066.00 |
| *Ditaxis biseriata* | 11174.00 | 1605.00 | 1315.00 | 791.00 | 1497.00 |
| *Chrysoperla nipponensis* | 11155.00 | 1497.00 | 1307.00 | 776.00 | 1244.00 |
| *Chrysopa pallens* | 11158.00 | 1572.00 | 1309.00 | 779.00 | 1905.00 |
| *Apochrysa matsumurae* | 11146.00 | 1500.00 | 1314.00 | 776.00 | 1409.00 |
| *Rapisma zayuanum* | 11164.00 | 1467.00 | 1316.00 | 777.00 | 1193.00 |
| *Sialis hamata* | 11192.00 | 1515.00 | 1321.00 | 784.00 | 814.00 |
| *Protohermes concolorus* | 11184.00 | 1479.00 | 1312.00 | 774.00 | 1131.00 |
| *Corydalus cornutus* | 11175.00 | 1504.00 | 1311.00 | 786.00 | 967.00 |
| *Neochauliodes punctatolosus* | 11166.00 | 1449.00 | 1318.00 | 789.00 | 1006.00 |
| *Mongoloraphidia harmandi* | 11074.00 | 1473.00 | 1312.00 | 788.00 | 1239.00 |
| Avg. | 11161.69 | 1504.31 | 1312.77 | 781.92 | 1203.31 |
