# Supplementary material for: Comparative Mitogenomic Analysis Reveals Sexual Dimorphism in a Rare Montane Lacewing (Insecta: Neuroptera: Ithonidae)
Source: PLoS One. 2013 Dec 31;8(12):e83986. doi: 10.1371/journal.pone.0083986 (PMC3877146; doi:10.1371/journal.pone.0083986)
Supplement: Table S3 — Organization of the Rapisma zayuanum mt genome. (DOC) [file pone.0083986.s003.doc]

**Table S3. Organization of** *Rapisma zayuanum* **mt genome**

| **Gene** | **Direction** | **Location (bp)** | **Size (bp)** | **Anticodon** | **Start Codon** | **Stop Codon** | **Intergenic**  **Nucleotide^*^** |
| --- | --- | --- | --- | --- | --- | --- | --- |
| *tRNA^Ile^* | J | 1-66 | 66 | 31-33 GAT |  |  | 0 |
| *tRNA^Gln^* | N | 96-164 | 69 | 132-134 TTG |  |  | 29 |
| *tRNA^Met^* | J | 191-260 | 70 | 221-223 CAT |  |  | 26 |
| *nad2* | J | 261-1274 | 1014 |  | ATT | TAA | 0 |
| *tRNA^Cys^* | N | 1273-1336 | 64 | 1304-1306 GCA |  |  | -2 |
| *tRNA^Trp^* | J | 1338-1403 | 66 | 1368-1370 TCA |  |  | 1 |
| *tRNA^Tyr^* | N | 1404-1470 | 67 | 1436-1438 GTA |  |  | 0 |
| *cox1* | J | 1471-3007 | 1537 |  | ATT | T-tRNA | 0 |
| *tRNA^Leu(UUR)^* | J | 3008-3072 | 65 | 3037-3039 TAA |  |  | 0 |
| *cox2* | J | 3079-3763 | 685 |  | ATG | T-tRNA | 6 |
| *tRNA^Lys^* | J | 3764-3834 | 71 | 3794-3796 CTT |  |  | 0 |
| *tRNA^Asp^* | J | 3834-3899 | 66 | 3862-3864 GTC |  |  | -1 |
| *atp8* | J | 3900-4058 | 159 |  | ATT | TAA | 0 |
| *atp6* | J | 4052-4726 | 675 |  | ATG | TAA | -7 |
| *cox3* | J | 4726-5514 | 789 |  | ATG | TAA | -1 |
| *tRNA^Gly^* | J | 5514-5578 | 65 | 5543-5545 TCC |  |  | -1 |
| *nad3* | J | 5579-5932 | 354 |  | ATT | TAA | 0 |
| *tRNA^Ala^* | J | 5940-6003 | 64 | 5969-5971 TGC |  |  | 7 |
| *tRNA^Arg^* | J | 6004-6069 | 66 | 6033-6035 TCG |  |  | 0 |
| *tRNA^Asn^* | J | 6069-6132 | 64 | 6099-6101 GTT |  |  | -1 |
| *tRNA^Ser(AGN)^* | J | 6133-6198 | 66 | 6154-6156 GCT |  |  | 0 |
| *tRNA^Glu^* | J | 6208-6277 | 70 | 6238-6240 TTC |  |  | 9 |
| *tRNA^Phe^* | N | 6276-6345 | 70 | 6310-6312 GAA |  |  | -2 |
| *nad5* | N | 6346-8071 | 1726 |  | ATA | T-tRNA | 0 |
| *tRNA^His^* | N | 8069-8135 | 67 | 8101-8103 GTG |  |  | -3 |
| *nad4* | N | 8136-9468 | 1333 |  | ATG | T-tRNA | 0 |
| *nad4l* | N | 9462-9749 | 288 |  | ATA | TAA | -7 |
| *tRNA^Thr^* | J | 9753-9818 | 66 | 9782-9784 TGT |  |  | 3 |
| *tRNA^Pro^* | N | 9819-9883 | 65 | 9851-9853 TGG |  |  | 0 |
| *nad6* | J | 9885-10403 | 519 |  | ATA | TAA | 1 |
| *cytb* | J | 10396-11532 | 1137 |  | ATG | TAA | -8 |
| *tRNA^Ser(UCN)^* | J | 11531-11597 | 67 | 11560-11562 TGA |  |  | -2 |
| *nad1* | N | 11617-12564 | 948 |  | TTG | TAG | 19 |
| *tRNA^Leu(CUN)^* | N | 12566-12628 | 63 | 11597-12599 TAG |  |  | 1 |
| *rrnL* | N | 12629-13944 | 1316 |  |  |  | 0 |
| *tRNA^Val^* | N | 13945-14014 | 70 | 13980-13982 TAC |  |  | 0 |
| *rrnS* | N | 14015-14791 | 777 |  |  |  | 0 |
| CR |  | 14792-15984 | 1193 |  |  |  | 0 |

“*”: Negative numbers indicate that adjacent genes overlap.
