# Supplementary material for: Comparative Mitogenomic Analysis Reveals Sexual Dimorphism in a Rare Montane Lacewing (Insecta: Neuroptera: Ithonidae)
Source: PLoS One. 2013 Dec 31;8(12):e83986. doi: 10.1371/journal.pone.0083986 (PMC3877146; doi:10.1371/journal.pone.0083986)
Supplement: Table S4 — Base composition and strand bias in the sequenced mt genomes of Neuroptera. (DOC) [file pone.0083986.s004.doc]

**Table S4. Base composition and strand bias in Neuropteran insect mt genomes**

| **Species** | **A%** | **T%** | **A+T%** | **AT-skew** | **C%** | **G%** | **C+G%** | **GC-skew** |
| --- | --- | --- | --- | --- | --- | --- | --- | --- |
| *Polystoechotes punctatus* | 38.340 | 40.610 | 78.960 | -0.030 | 12.220 | 8.820 | 21.040 | -0.160 |
| *Libelloides macaronius* | 39.930 | 34.570 | 74.500 | 0.070 | 15.000 | 10.500 | 25.500 | -0.180 |
| *Ascaloptynx appendiculatus* | 40.340 | 35.230 | 75.570 | 0.070 | 14.730 | 9.700 | 24.430 | -0.210 |
| *Ditaxis biseriata* | 40.520 | 39.280 | 79.790 | 0.020 | 11.920 | 8.290 | 20.210 | -0.180 |
| *Chrysoperla nipponensis* | 39.170 | 39.710 | 78.890 | -0.010 | 12.010 | 9.100 | 21.110 | -0.140 |
| *Apochrysa matsumurae* | 38.070 | 40.950 | 79.020 | -0.040 | 12.360 | 8.620 | 20.980 | -0.180 |
| *Rapisma zayuanum* | 37.810 | 43.280 | 81.090 | -0.070 | 11.200 | 7.710 | 18.910 | -0.180 |
| *Chrysopa pallens* | 39.920 | 39.630 | 79.550 | 0.004 | 11.710 | 8.740 | 20.450 | -0.150 |
| *Sialis hamata* | 39.730 | 38.590 | 78.320 | 0.010 | 12.690 | 8.980 | 21.680 | -0.170 |
| *Protohermes concolorus* | 37.490 | 38.340 | 75.830 | -0.010 | 15.150 | 9.020 | 24.170 | -0.250 |
| *Corydalus cornutus* | 37.970 | 36.920 | 74.900 | 0.010 | 15.840 | 9.260 | 25.100 | -0.260 |
| *Neochauliodes punctatolosus* | 38.820 | 37.550 | 76.370 | 0.020 | 14.760 | 8.870 | 23.630 | -0.250 |
| *Mongoloraphidia harmandi* | 41.080 | 39.230 | 80.310 | 0.020 | 12.090 | 7.600 | 19.690 | -0.230 |
| Avg. | 39.168 | 38.761 | 77.931 | 0.005 | 13.206 | 8.862 | 22.069 | -0.195 |
