# Supplementary material for: Comparative Mitogenomic Analysis Reveals Sexual Dimorphism in a Rare Montane Lacewing (Insecta: Neuroptera: Ithonidae)
Source: PLoS One. 2013 Dec 31;8(12):e83986. doi: 10.1371/journal.pone.0083986 (PMC3877146; doi:10.1371/journal.pone.0083986)
Supplement: Table S5 — Codon usage of protein-coding genes in the Rapisma zayuanum mt genome. (DOC) [file pone.0083986.s005.doc]

**Table S5. Codon usage of protein-coding genes in the** ***Rapisma zayuanum*** genome

| **AA** | **Codon*** | **n** | **%** | **RSCU** | **AA** | **Codon** | **n** | **%** | **RSCU** |
| --- | --- | --- | --- | --- | --- | --- | --- | --- | --- |
| Phe | UUU(F) | 341 | 9.19 | 1.88 | Ser | UCU(S) | 115 | 3.10 | 2.68 |
|  | UUC(F) | 22 | 0.59 | 0.12 |  | UCC(S) | 5 | 0.13 | 0.12 |
| Leu | UUA(L) | 529 | 14.25 | 5.21 |  | UCA(S) | 95 | 2.56 | 2.22 |
|  | UUG(L) | 17 | 0.46 | 0.17 |  | UCG(S) | 1 | 0.03 | 0.02 |
|  | CUU(L) | 41 | 1.10 | 0.40 | Ser(s) | AGU(S) | 85 | 2.29 | 2.74 |
|  | CUC(L) | 1 | 0.03 | 0.01 |  | AGC(S) | 5 | 0.13 | 0.16 |
|  | CUA(L) | 20 | 0.54 | 0.20 |  | AGA(S) | 33 | 0.89 | 1.06 |
|  | CUG(L) | 1 | 0.03 | 0.01 |  | AGG(S) | 1 | 0.03 | 0.03 |
| Ile | AUU(I) | 437 | 11.78 | 1.93 | Thr | ACU(T) | 93 | 2.51 | 2.28 |
|  | AUC(I) | 15 | 0.40 | 0.07 |  | ACC(T) | 10 | 0.27 | 0.25 |
| Met | AUA(M) | 218 | 5.87 | 1.88 |  | ACA(T) | 57 | 1.54 | 1.40 |
|  | AUG(M) | 14 | 0.38 | 0.12 |  | ACG(T) | 3 | 0.08 | 0.07 |
| Val | GUU(V) | 69 | 1.86 | 2.28 | Ala | GCU(A) | 83 | 2.24 | 2.50 |
|  | GUC(V) | 1 | 0.03 | 0.03 |  | GCC(A) | 9 | 0.24 | 0.27 |
|  | GUA(V) | 47 | 1.27 | 1.55 |  | GCA(A) | 40 | 1.08 | 1.20 |
|  | GUG(V) | 4 | 0.11 | 0.13 |  | GCG(A) | 1 | 0.03 | 0.03 |
| Tyr | UAU(Y) | 163 | 4.39 | 1.82 | Cys | UGU(C) | 34 | 0.92 | 1.94 |
|  | UAC(Y) | 16 | 0.43 | 0.18 |  | UGC(C) | 1 | 0.03 | 0.06 |
|  | UAA(*) | 0 | 0.00 | 0.00 | Trp | UGA(W) | 94 | 2.53 | 1.90 |
|  | UAG(*) | 0 | 0.00 | 0.00 |  | UGG(W) | 5 | 0.13 | 0.10 |
| His | CAU(H) | 71 | 1.91 | 1.95 | Arg | CGU(R) | 23 | 0.62 | 1.67 |
|  | CAC(H) | 2 | 0.05 | 0.05 |  | CGC(R) | 1 | 0.03 | 0.07 |
| Gln | CAA(Q) | 64 | 1.72 | 2.00 |  | CGA(R) | 29 | 0.78 | 2.11 |
|  | CAG(Q) | 0 | 0.00 | 0.00 |  | CGG(R) | 2 | 0.05 | 0.15 |
| Asn | AAU(N) | 216 | 5.82 | 1.90 | Pro | CCU(P) | 36 | 0.97 | 0.84 |
|  | AAC(N) | 11 | 0.30 | 0.10 |  | CCC(P) | 2 | 0.05 | 0.05 |
| Lys | AAA(K) | 93 | 2.51 | 1.88 |  | CCA(P) | 87 | 2.34 | 2.03 |
|  | AAG(K) | 6 | 0.16 | 0.12 |  | CCG(P) | 2 | 0.05 | 0.05 |
| Asp | GAU(D) | 64 | 1.72 | 1.88 | Gly | GGU(G) | 63 | 1.70 | 1.29 |
|  | GAC(D) | 4 | 0.11 | 0.12 |  | GGC(G) | 1 | 0.03 | 0.02 |
| Glu | GAA(E) | 74 | 1.99 | 1.95 |  | GGA(G) | 108 | 2.91 | 2.20 |
|  | GAG(E) | 2 | 0.05 | 0.05 |  | GGG(G) | 24 | 0.65 | 0.49 |

“*”: A total of 3711 codons from *Rapisma zayuanum* are analyzed, excluding the start and stop codons. AA, amino acid; RSCU, Relative synonymous codon usage; n = frequency of each codon. % = n/3711.
