# Supplementary material for: Comparative Mitogenomic Analysis Reveals Sexual Dimorphism in a Rare Montane Lacewing (Insecta: Neuroptera: Ithonidae)
Source: PLoS One. 2013 Dec 31;8(12):e83986. doi: 10.1371/journal.pone.0083986 (PMC3877146; doi:10.1371/journal.pone.0083986)
Supplement: Table S6 — Base composition and strand bias in PCGs of Rapisma zayuanum . (DOC) [file pone.0083986.s006.doc]

**Table S6. Base composition and strand bias in PCGs of** ***Rapisma zayuanum.***

| **Protein** | **A** | **T** | **A+T** | **AT-skew** | **C** | **G** | **C+G** | **GC-skew** |
| --- | --- | --- | --- | --- | --- | --- | --- | --- |
| *atp6* | 31.85 | 47.11 | 78.96 | -0.19 | 11.41 | 9.63 | 21.04 | -0.08 |
| *atp8* | 38.36 | 50.31 | 88.68 | -0.13 | 8.81 | 2.52 | 11.32 | -0.56 |
| *cox1* | 28.95 | 44.11 | 73.06 | -0.21 | 13.73 | 13.21 | 26.94 | -0.02 |
| *cox2* | 32.85 | 43.07 | 75.91 | -0.13 | 13.28 | 10.80 | 24.09 | -0.10 |
| *cox3* | 31.43 | 44.36 | 75.79 | -0.17 | 12.67 | 11.53 | 24.21 | -0.05 |
| *cytb* | 30.96 | 45.47 | 76.43 | -0.19 | 13.81 | 9.76 | 23.57 | -0.17 |
| *nad1* | 33.23 | 45.46 | 78.69 | -0.16 | 7.59 | 13.71 | 21.31 | 0.29 |
| *nad2* | 30.67 | 52.37 | 83.04 | -0.26 | 9.27 | 7.69 | 16.96 | -0.09 |
| *nad3* | 32.20 | 48.87 | 81.07 | -0.21 | 11.30 | 7.63 | 18.93 | -0.19 |
| *nad4* | 36.08 | 45.84 | 81.92 | -0.12 | 6.30 | 11.78 | 18.08 | 0.30 |
| *nad4l* | 35.07 | 47.22 | 82.29 | -0.15 | 5.90 | 11.81 | 17.71 | 0.33 |
| *nad5* | 37.31 | 45.08 | 82.39 | -0.09 | 6.84 | 10.78 | 17.61 | 0.22 |
| *nad6* | 34.49 | 50.87 | 85.36 | -0.19 | 8.86 | 5.78 | 14.64 | -0.21 |
| Avg. | 33.34 | 46.93 | 80.28 | -0.17 | 9.98 | 9.74 | 19.72 | -0.03 |
