# Supplementary material for: Comparative Mitogenomic Analysis Reveals Sexual Dimorphism in a Rare Montane Lacewing (Insecta: Neuroptera: Ithonidae)
Source: PLoS One. 2013 Dec 31;8(12):e83986. doi: 10.1371/journal.pone.0083986 (PMC3877146; doi:10.1371/journal.pone.0083986)
Supplement: Table S7 — Evolution rates of the 13 PCGs of eight species of Neuroptera. (DOC) [file pone.0083986.s007.doc]

**Table S7. Evolutionary rates of the 13 PCGs of Neuroptera.**

| Species | *atp6* | *atp8* | *cox1* | *cox2* | *cox3* | *cytb* | *nad1* | *nad2* | *nad3* | *nad4* | *nad4l* | *nad5* | *nad6* |
| --- | --- | --- | --- | --- | --- | --- | --- | --- | --- | --- | --- | --- | --- |
| *Apochrysa matsumurae* | 0.18 | 0.34 | 0.08 | 0.11 | 0.15 | 0.20 | 0.24 | 0.46 | 0.20 | 0.29 | 0.49 | 0.37 | 0.51 |
| *Ascaloptynx appendiculatus* | 0.14 | 0.68 | 0.08 | 0.09 | 0.11 | 0.06 | 0.19 | 0.34 | 0.22 | 0.36 | 0.29 | 0.31 | 0.32 |
| *Chrysopa pallens* | 0.18 | 0.71 | 0.08 | 0.10 | 0.11 | 0.20 | 0.31 | 0.34 | 0.35 | 0.35 | 0.78 | 0.47 | 0.78 |
| *Chrysoperla nipponensis* | 0.20 | 0.47 | 0.08 | 0.12 | 0.10 | 0.17 | 0.25 | 0.40 | 0.40 | 0.34 | 0.37 | 0.41 | 0.46 |
| *Ditaxis biseriata* | 0.15 | 0.36 | 0.07 | 0.09 | 0.08 | 0.15 | 0.20 | 0.52 | 0.19 | 0.40 | 0.40 | 0.36 | 0.58 |
| *Rapisma zayuanum* | 0.15 | 0.77 | 0.08 | 0.15 | 0.14 | 0.17 | 0.26 | 0.40 | 0.23 | 0.36 | 0.49 | 0.30 | 0.48 |
| *Polystoechotes punctatus* | 0.12 | 0.56 | 0.05 | 0.08 | 0.06 | 0.08 | 0.17 | 0.35 | 0.23 | 0.36 | 0.47 | 0.32 | 0.43 |
