# Supplementary material for: Comparative Mitogenomic Analysis Reveals Sexual Dimorphism in a Rare Montane Lacewing (Insecta: Neuroptera: Ithonidae)
Source: PLoS One. 2013 Dec 31;8(12):e83986. doi: 10.1371/journal.pone.0083986 (PMC3877146; doi:10.1371/journal.pone.0083986)
Supplement: Table S8 — Genetic distance of the three montane lacewing species based on rrnL . (DOC) [file pone.0083986.s008.doc]

**Table S8. Genetic distance of the three montane lacewing species based on *rrnL*.**

| Species | *Rapisma zayuanum* | *Rapisma xizangense* | *Rapisma cryptunum* |
| --- | --- | --- | --- |
| *Rapisma zayuanum* | - |  |  |
| *Rapisma xizangense* | 0.0000 | - |  |
| *Rapisma cryptunum* | 0.0829 | 0.0829 | - |
