# Supplementary material for: Comparative Mitogenomic Analysis Reveals Sexual Dimorphism in a Rare Montane Lacewing (Insecta: Neuroptera: Ithonidae)
Source: PLoS One. 2013 Dec 31;8(12):e83986. doi: 10.1371/journal.pone.0083986 (PMC3877146; doi:10.1371/journal.pone.0083986)
Supplement: Table S9 — Genetic distance between Rapisma zayuanum and Rapisma xizangense based on PCGs and rRNAs. (DOC) [file pone.0083986.s009.doc]

**Table S9. Genetic distance between *Rapisma zayuanum* and *Rapisma xizangense* based on PCGS and rRNAs.**

| **Genes** | **K2P-distance** | **p-distance** |
| --- | --- | --- |
| *rrnS* | 0.0026 | 0.0026 |
| *rrnL* | 0.0015 | 0.0015 |
| *atp6* | 0.0060 | 0.0060 |
| *atp8* | 0.0000 | 0.0000 |
| *cox1* | 0.0013 | 0.0013 |
| *cox2* | 0.0015 | 0.0015 |
| *cox3* | 0.0051 | 0.0051 |
| *cytb* | 0.0062 | 0.0062 |
| *nad1* | 0.0011 | 0.0011 |
| *nad2* | 0.0030 | 0.0030 |
| *nad3* | 0.0057 | 0.0057 |
| *nad4* | 0.0045 | 0.0045 |
| *nad4l* | 0.0000 | 0.0000 |
| *nad5* | 0.0041 | 0.0041 |
| *nad6* | 0.0058 | 0.0058 |
| Avg. | 0.0032 | 0.0032 |
