# Supplementary material for: Comparative Mitogenomic Analysis Reveals Sexual Dimorphism in a Rare Montane Lacewing (Insecta: Neuroptera: Ithonidae)
Source: PLoS One. 2013 Dec 31;8(12):e83986. doi: 10.1371/journal.pone.0083986 (PMC3877146; doi:10.1371/journal.pone.0083986)
Supplement: Table S10 — Primers used in this study. (DOC) [file pone.0083986.s010.doc]

**Table S10.** Primer sequences used in this study

| **No. fragment^*^** | **Primer ID** | **Nucleotide sequence (5’-3’)** | **Reference** |
| --- | --- | --- | --- |
| 1 | N2-J586 | CCATTCCATTTYTGATTTCC | Simon *et al.*, 2006 |
|  | C1- N1738 | TTTATTCGTGGAAATGCTATGTC | Simon *et al.*, 2006 |
| 2 | F-1738 | TCAGGGTTAATTGGAACTAG | Present study |
|  | R-2756 | GGGTAAGATTGTAATCATTC | Present study |
| 3 | C1-J2756 | ACATTTTTTCCTCAACATTT | Simon *et al.*, 2006 |
|  | C2-N3665 | CCACAAATTTCTGAACACTG | Simon *et al.*, 2006 |
| 4 | F-3665 | GATAATCGAGCAATTTTACC | Present study |
|  | R-3790 | AGTAATAGGGTCAAATATTG | Present study |
| 5 | TK-J3790 | CATTAGATGACTGAAAGCAAGTA | Simon *et al.*, 2006 |
|  | A6-N4552 | ATGGTCWGCAATYATATTWGC | Simon *et al.*, 2006 |
| 6 | F-4552 | GATTCCTCAAATAAGTCCTC | Present study |
|  | R-6172 | GTGCTGGTTATATAATTCAT | Present study |
| 7 | TN-J6172 | AGAGGCAATTTATTGTTAATAA | Simon *et al.*, 2006 |
|  | N5-N7211 | TTAAGGCTTTATTATTTATATGTGC | Simon *et al.*, 2006 |
| 8 | N5-J7077 | TTAAATCCTTWGARTAAAAYCC | Simon *et al.*, 2006 |
|  | N5-N7793 | TTAGGTTGRGATGGNYTAGG | Simon *et al.*, 2006 |
| 9 | N5-J7572 | AAAGGGAATTTGAGCTCTTTTWGT | Simon *et al.*, 2006 |
|  | N4-N8727 | AAATCTTTRATTGCTTATTCWTC | Simon *et al.*, 2006 |
| 10 | N4-J8641 | CCAGAAGAACACAAACCATG | Simon *et al.*, 2006 |
|  | N4L-N9629 | GTTTGTGAGGGTGCAATAGG | Simon *et al.*, 2006 |
| 11 | F-9629 | ATTCTTTCTCTAGCTATTAG | Present study |
|  | R-9648 | TCCTAAAGATAAAGGATGAT | Present study |
| 12 | N4L-J9648 | ACCTAAAGCTCCCTCACAWAC | Simon *et al.*, 2006 |
|  | CB-N11010 | TATCTACAGCRAATCCYCCYCA | Simon *et al.*, 2006 |
| 13 | CB-J11335 | CATATTCAACCWGAATGRTA | Simon *et al.*, 2006 |
|  | N1-N12067 | AATCGWACTCCWTTTGATTTTGC | Simon *et al.*, 2006 |
| 14 | N1-J11876 | CGAGGTAAAGTMCCWTTTGATTTTGC | Simon *et al.*, 2006 |
|  | N1-N12595 | GTWGCTTTTTTAACTTTATTRGARCG | Simon *et al.*, 2006 |
| 15 | N1-J12261 | AACTTCATAAGAAATAGTYTGRGC | Simon *et al.*, 2006 |
|  | LR-N13000 | TTACCTAGGGATAACAGCGTAA | Simon *et al.*, 2006 |
| 16 | LR-J12888 | CCGGTCTGAACTCAGATCATGTA | Simon *et al.*, 2006 |
|  | LR-N13889 | ATTTATTGTACCTTTTGTATCAG | Simon *et al.*, 2006 |
| 17 | LR-J13342 | CCTTAGCACAGTTAAAATACTGC | Simon *et al.*, 2006 |
|  | LR-N14220 | TTATGCACATATCGCCCGTC | Simon *et al.*, 2006 |
| 18 | LR-J14197 | GTAAAYCTACTTTGTTACGACTT | Simon *et al.*, 2006 |
|  | SR-N14745 | GTGCCAGCAAYCGCGGTTATAC | Simon *et al.*, 2006 |
| 19 | F-14745 | AAGTGCCTGAATTAAAGGAT | Present study |
|  | R-586 | TCGAGGTATTCCTCTTAAAC | Present study |

“*”: The orientation is shown in Figure 1.
